# A critique of the English national policy from a social determinants of health perspective using a realist and problem representation approach: the ‘Childhood Obesity: a plan for action’ (2016, 2018, 2019)

**DOI:** 10.1186/s12889-021-12364-6

**Published:** 2021-12-14

**Authors:** Naomi Griffin, Sophie M. Phillips, Frances Hillier-Brown, Jonathan Wistow, Hannah Fairbrother, Eleanor Holding, Katie Powell, Carolyn Summerbell

**Affiliations:** 1 Fuse, the NIHR (UK) Centre for Translational Research in Public Health (website), Newcastle, UK; 2 Department of Sport and Exercise Sciences, Durham University, Durham, UK; 3 Population Health Sciences Institute, Newcastle University, Newcastle, UK; 4 Department of Sociology, Durham University, Durham, UK; 5 Health Sciences School, University of Sheffield, Sheffield, UK; 6 ScHARR, School of Health and Related Research, University of Sheffield, Sheffield, UK

**Keywords:** Childhood Obesity, Health inequalities, Social Determinants of Health, Health Policy

## Abstract

**Background:**

The UK government released Chapter 1 of the ‘Childhood Obesity: a plan for action’ (2016), followed by Chapter 2 (2018) and preliminary Chapter 3 was published for consultation in 2019 (hereon collectively ‘*The Policy’)*. The stated policy aims were to reduce the prevalence of childhood obesity in England, addressing disparities in health by reducing the gap *(approximately two-fold)* in childhood obesity between those from the most and least deprived areas.

**Methods:**

Combining a realist approach with an analysis of policy discourses, we analysed the policies using a social determinants of health (SDH) perspective (focusing on socio-economic inequalities). This novel approach reveals how the framing of policy ‘problems’ leads to particular approaches and interventions.

**Results:**

While recognising a social gradient in relation to obesity measures, we critique obesity problem narratives. *The Policy* included some upstream, structural approaches (e.g. restrictions in food advertising and the soft-drinks industry levy). However, the focus on downstream individual-level behavioural approaches to reduce calorie intake and increase physical activity does not account for the SDH and the complexity and contestedness of ‘obesity’ and pays insufficient attention to how proposals will help to reduce inequalities. Our findings illustrate that individualising of responsibility to respond to what wider evidence shows is structural inequalities, can perpetuate damaging narratives and lead to ineffective interventions, providing caution to academics, practitioners and policy makers (local and national), of the power of problem representation. Our findings also show that the problem framing in *The Policy* risks reducing important public health aims to encourage healthy diets and increase opportunities for physical activity (and the physical and mental health benefits of both) for children to weight management with a focus on particular children.

**Conclusions:**

We propose an alternative conceptualisation of the policy ‘problem’, that obesity rates are illustrative of inequality, arguing there needs to be policy focus on the structural and factors that maintain health inequalities, including poverty and food insecurity. We hope that our findings can be used to challenge and strengthen future policy development, leading to more effective action against health inequalities and intervention-generated inequalities in health.

**Supplementary Information:**

The online version contains supplementary material available at 10.1186/s12889-021-12364-6.

## Background

Childhood obesity has been identified as a public health priority in high income countries across the world [1]. In response, countries have developed national and local policies, and have implemented multiple public health interventions, to try and tackle the problem [1]. Taking England as an example, childhood obesity has been identified as a policy priority since 1991 [2]. Jebb and colleagues [3] described the evolution of policy and actions to tackle obesity in England up to 2013, concluding that rigorous evaluations of effectiveness were rare, and that the limited evidence of tangible success, despite substantial investment of resources, reinforces the magnitude of the challenge to the whole of society. More recently, Theis and White [4] analysed English government obesity policies using theoretical frameworks and an intensive applied thematic analysis approach. The analysis revealed that National obesity policy proposals rely heavily on individual level behaviour, are repeated with no reference to previous policies, and are proposed with limited guidance on implementation. Croker and colleagues [5] conducted a mapping study of national policies for pre-school children obesity in England from a behavioural science perspective. They found that much of the policy activity is focussed on education and suggested that upstream policies which act on food systems should be strengthened. Although the importance of the socioeconomic patterning of childhood obesity is acknowledged in these existing analyses of policy, it was not the focus of their analyses.

The recent 2019 Chief Medical Officers report *Time to Solve Childhood Obesity* [6] underlines the importance of the social determinants of health in understanding childhood obesity rates. The most recent national policy for tackling childhood obesity in England has been published as chapters, first in 2016 ‘Childhood Obesity: a plan for action’ [7], followed by ‘Childhood Obesity: a plan for action: Chapter 2’ in 2018 [8]. A preliminary Chapter 3 was opened for consultation in 2019 in the green paper ‘Advancing our health: prevention in the 2020s’ [9]. For convenience the chapters will collectively be referred to in this paper as *The Policy*. The stated aim of *The Policy* was to significantly reduce the prevalence of childhood obesity in England, and to address disparities in health by reducing the gap in childhood obesity between those from the most and least deprived areas. Although the creation of a policy addressing childhood obesity was generally welcomed by public health bodies at the time of publication, there is concern that they focus too heavily on individual behaviour change rather than upstream (stealth) interventions [4, 5, 10, 11]. With plans underway for Chapter 3 of *The Policy* [9] potentially delayed due to a Government focus on Covid-19, it is our hope that government will revisit and review the aims of *The Policy* with a focus on the structural influences of health inequalities and poverty on health outcomes.

### Childhood obesity and inequalities in England

The relationship between social disadvantage and health is well documented (see for example: [12–16]). There is a social gradient that can be mapped onto childhood obesity data (as measured by Body Mass Index) [14] with higher prevalence seen in children from areas of higher socio-economic deprivation [17, 18]. This pattern has been evidenced globally [1]. Children living in the most deprived areas in England are twice as likely to measure as ‘obese’ than children in the most affluent groups [19], and the gap between the most and least deprived is growing with a plateauing of prevalence for the most advantaged [14].

### Social determinants of health

A social determinants of health (SDH) perspective explores how individual experience of health is affected by micro and macro social and political contexts which lead to health inequalities [15]. The Marmot Review [13] was critical in the development of SDH perspectives designed to shape policy in England which emphasised the importance of the ‘causes of the causes’ of health and health inequalities. The review argues that ‘*health is closely linked to the conditions in which people are born, grow, live, work and age and inequities in power, money and resources – the social determinants of health’* ([13], p5). Early years; education; work; income; and communities were identified as key examples of where the social gradient in health is persistent [13]. Health policy has been critiqued for neglecting structural forces as causal factors in producing social and economic inequalities and health inequalities (e.g. [13, 14, 20]). Even when the social determinants of health are acknowledged in policy and policy networks, structural factors that create and sustain inequalities are often not meaningfully addressed [15].

### Childhood obesity policy context and the social determinants of health

Traditionally, policy discourses around obesity have focused on personal responsibility and individualism, with an absence of engagement with the social determinants of health [20, 21]. Individualistic approaches are evident in the wealth of research examining risk factors for childhood obesity, which focus on implementing changes to lifestyle behaviours [11] with minimal consideration of the wider social determinants of health. A pattern in approaches to obesity interventions lacking complexity has also been found at a local authority level in England [22]. Existing systematic reviews of the effectiveness of interventions to prevent childhood obesity which focus on health inequalities [23, 24] found that most interventions did not report their results by socio-economic status nor used a social determinants of health approach to intervention development or implementation. There is often little mention of economic, cultural and social issues in relation to obesity and where wider determinants such as socioeconomic status, food insecurity, or education level are mentioned, behavioural and lifestyle modifications are still prioritised [20, 21]. This is despite the UK having one of the highest levels of children living in severely food insecure households in Europe [25], and evidence that austerity and budget cuts have negatively impacted Local Authority capacity to reduce health inequalities [26].

The aim of our research was to analyse *The Policy* using a social determinants of health (SDH) perspective. Our findings can then be used to challenge and strengthen future policy development, leading to more effective action against health inequalities and intervention-generated inequalities in health. The Covid-19 pandemic has further exposed social gradients in health, with those experiencing poverty and disadvantage being hit hardest [27], and worsening food insecurity [28]. Food bank use has significantly increased; Trussell Trust [29] reported that food bank use had increased by 74% over the past 5 years, with 1.9 million emergency food supplies delivered to individuals across the UK between April 2019-March 2020, and 700,000 of these parcels went to children. It is critical that consideration is given to these issues in future policy proposals [30, 31]. With plans underway for Chapter 3 of *The Policy* [9] potentially delayed due to a Government focus on Covid-19, it is our hope that government will revisit and review the aims of *The Policy* with a focus on health inequalities and poverty, using a stronger critical structural lens.

## Methods

We used a novel methodological approach, employing and integrating Pawson and Tilley’s [32] realistic evaluation with Bacchi’s ‘What’s the Problem Represented to be?’ (WPR) approach [33, 34] to analyse *The Policy* [7–9]. Pawson and Tilley’s [32] realist approach was used to understand the proposed pathways for reducing inequalities, assessing the inherent ‘programme theories’ within *The Policy*: what the policy proposes to do and the intended results, and what the (sometimes implied) pathways to said results are, and how success will be measured. These proposed pathways were then assessed based on how embedded they were in the realities of policy implementation and how they take account of external factors on policy processes. Bacchi’s WPR approach [33] was used to analyse government and external discourses around *The Policy*, aiming to uncover how policy ‘problems’ are discursively created within policy documents through the way ‘problems’ are represented. This approach asks the researcher to start with policy proposals and reflect on what the proposals imply that ‘the problem’ is (e.g., a proposal to increase training implies a lack of training to be the problem). Importantly, the way policy ‘problems’ are discursively produced can also set the parameters for the discourse that follows [33, 34]. In this way, the WPR tool affords a productive means of identifying and interrogating the power of narratives that may otherwise be taken for granted. Our methodology acknowledges that all policy documents contribute to and are informed by wider narratives which frame proposals and interventions and therefore interrogating said narratives can help to understand their effects. Table 1 provides an overview of the criteria used to extract data from the policy documents.

**Table 1:** Data extraction criteria

Realist review - Pawson and Tilley [32]	Problem representation – Bacchi [33]
• **Programme theory** o Identification of the programme theory as a basis for understanding the intended policy pathway	• **What is the ‘problem’ represented to be?** o How does the policy represent the problem under investigation? o How has this representation come about? o ‘The problem’ can be inferred from the proposed ‘solution’.
• **Embedded** o How (if at all) does the policy conceptualise the social systems in which the policies are being delivered? • **Open systems** o How (if at all) are externalities understood, identified and addressed in the policy? • **Agency** o How (if at all) is agency understood, identified and addressed in the policy?	• **Assumptions underpinning the representation of the problem** o What are the presuppositions/ assumptions that underlie the representation and its concepts and categories? • **Effects of problem representation** o What effects are produced by this representation of the ‘problem’?
• **Inequalities focus** o How are inequalities addressed in the policy? What inequalities are addressed? How so the proposed pathways to change claim to reduce inequalities?	• **What is left unproblematic and how might the policy response differ** o What is left unproblematic in this problem representation? Where are the silences? o Can the ‘problem’ be conceptualized differently? o What is left out of the problem representation?

### Data extraction and analysis

*The Policy* documents were independently double data extracted between October 2019 and May 2020. Researchers with different academic backgrounds extracted and analysed the policies (NG & SMP), to allow broader identification and interpretation, and to enable a more diverse discussion of the findings [35]. We developed a coding framework based on the questions in Table 1, then extracted data from the policy documents using coding software. The two researchers carried out data extraction of policy documents separately, then brought together the extracted data to identify any differences or disagreements through several data extraction and analysis meetings, moderated by a 3^rd^ researcher (FHB). All authors were then given the opportunity to comment on findings and analysis at several stages of the analysis and writing process.

## Findings

Our findings begin by outlining the key proposals (see supplementary Table S1), the proposed pathways to change from the proposals, the proposed measurements and what they tell us about the policy aims and scope. Then the framing of the ‘problem’ (in Bacchi’s sense [33, 34]) is discussed in depth, drawing from wider evidence to illustrate the framing of the policy ‘problem’ of obesity in the context of wider research that illustrates complexity and contested nature of the topic (see Supplementary Table S2 for examples of ‘problems’ as represented in *The Policy*). We then discuss the policy’s approach to inequalities, highlighting fundamental gaps in between proposed aims to reduce inequalities in child health and the proposed pathways to do this. Situating the problem representation in *The Policy* within a context of policy absences and alternative conceptualisations illustrates the effects of problem framing, allowing for the re-imagining of policy approaches to, and discourses around, the public health priority of ‘childhood obesity’ and its relationship with inequalities.

### Reviewing the key policy proposals

*The Policy* outlines that it is a response to the growing prevalence of childhood obesity (as measured by BMI) in England. *The Policy* states that the rising level of childhood obesity will result in rising obesity levels in adulthood that will cause other associated health problems, increasing chronic disease related to obesity (targeting an anticipated threat). *The Policy* predicts that this link will result in greater long-term cost to the NHS for obesity related health problems. Morbidities that have been linked to obesity (particularly type 2 diabetes) in adulthood and the link between obesity in childhood and adulthood are given to justify the policy’s pertinence, proposing to reduce the cost to the NHS by reducing the risk of health problems associated with obesity in adulthood through obesity reduction in children. *The Policy* proposals (Table S1) imply that behaviour change and reduction in obesity and child health inequalities will follow from the proposals.

The key proposals in *The Policy* suggest that it will tackle obesity through lowering sugar consumption, the reformulation of products and increase physical activity, and (after consultation and publication of Chapter 2) reducing promotion and advertising of unhealthy food and drink. An overview of the key proposals in *The Policy* (see Table S1) indicate that despite the different system levels that *The Policy* proposals cover the focus of proposals in on individual behaviour change without adequate engagement with wider determinants. Although the implementation of ‘upstream’ approaches such as the sugar tax and financial support in the case of the Healthy Start Scheme (HSS) are welcomed, *The Policy* focuses heavily on individual choice and behaviour (particularly of parents). Our findings support those of Chapman et al. ([36], p.20) that *The Policy* ‘*replicated a wider trend in which only aspirations for individual-level behaviours were articulated with precision*.’

Due perhaps to the brevity of the policy documents, how the impact of the policies listed in the proposal will be measured beyond the National Child Measurement Programme (see further discussion below) is unclear. For example, the measurement of mandatory calorie labelling, TV advertising restrictions, and local area changes is not outlined, which makes assessing the pathways to impact difficult. Ofsted are granted responsibility for tracking progress in schools. The ‘Sugar Tax’ is being monitored by industry responses, but it is not clear how directly the impact will be measured in terms of obesity prevalence. There is limited engagement with external influences on impact and implementation of the policy proposals, and its successes, supporting the findings of Theis and White ([4], p126) that the proposals do ‘not readily lead to implementation’.

### What’s the problem represented to be? Defining the ‘childhood obesity’ policy problem

*The Policy*’s definition of ‘obesity’ focuses on child weight status where the determinants of change are physical activity levels and calorie intake (i.e., calories consumed vs energy expended): ‘*at its root obesity is caused by an energy imbalance: taking in more energy through food than we use through activity*’ ([7], p.3). However, the causes of ‘obesity’ (as defined by BMI) are embedded in an extremely complex biological system that interact with cultural, structural and economic contextual factors, none of which exist in isolation [37]. Systemic factors such as money, power and resources are necessary for understanding the social gradient seen in obesity data [38]. The focus of energy balance at an individual level does not acknowledge the complex and contested nature of causes, its contested relationship to health, and how ‘obesity’ is defined and measured, within wider public health research (see for example, [39–42]). Therefore, explanations of BMI data which rely on individual energy imbalance must be challenged.

A narrow definition of obesity is also reflected in the key measure highlighted in the policy documents being the National Child Measurement Programme (NCMP) which is based on BMI (body mass index). Measures which rely on BMI (designed for use in adults), and the NCMP in particular [41–43], have been criticised for simplicity and for generalising a relationship between weight and health (see for example [44, 45]). Such a measure implies a definition of obesity which is not about the presence of illness or health problems, instead categorising individuals as overweight or obese based simply on height and weight [41, 44]. BMI is not a measure of *overall* health and thus the limitations of BMI (and any such screening method) and its complex association with health needs to be acknowledged. The tracking of childhood obesity as measured by BMI into adulthood (stated as a reason for the need to tackle childhood obesity) is also not clear cut. Increased likelihood of obesity in adulthood is apparent in those with obesity in childhood and adolescence; however, a high proportion (70%) of adults that fall into the obesity category did not in childhood or adolescence [46]. Evidence has suggested an association between childhood obesity (as measured by BMI) and later adult morbidity (e.g cardiovascular disease and metabolic health risks); however, this is far from conclusive, and the nature of the relationship is unclear [47, 48]. The combining of, and interchangeable use of, ‘obesity’ and ‘overweight’ in the policy also paints a misleading picture as morbidity correlation and risk differs between the categories. Where complexity and contextual factors are absent in policy proposals and the measurement of policy outcomes, it is implied that they are not relevant to understanding the policy ‘problem’.

### The effects of problem framing

Obesity is framed as an avoidable financial cost to health services in *The Policy* which perpetuates a ‘burden’ narrative [38]. It is worth recognising that individuals (the general public) have little control over how resources are distributed and budgets allocated within health systems. Difficult decisions on where to invest in public health often need to be made, especially where resources are scarce, and preference can swing to the treatment of ‘identifiable victims’ rather than investment in long-term prevention activities [49]. There is also a notable absence of the impact of austerity on health budgets and spending and child health inequalities in the policy documents, even when referring to inequalities and poverty, despite links made between poverty and childhood obesity. The absence of the impact of austerity on NHS and local public health budgets in *The Policy* purports a narrative that focuses on individual responsibility rather than a service provision issue (i.e. those that require healthcare are a ‘burden’ on limited resources rather than that there is a resourcing issue that is negatively impacting individuals requiring healthcare). Focussing on the individual (or parents) as responsible for making changes to childhood obesity levels contributes to a narrative of blame [50] that does not account for structural inequalities and social determinants of health beyond individual control [21]. Individual blame narratives, then, work to further justify a focus on individual level behaviour change in policy rather than a focus on the SDH which can explain the gradient in BMI population data relative to socio-economic deprivation.

Stigma was given as a reason for the need for a childhood obesity policy, as children deemed overweight or obese are likely to experience ‘*bullying, stigmatization and low self-esteem*' ([8] p6). However, as there was no targeted response to stigma itself. In reviewing the literature, the attention paid to stigma is necessary. The physical and psychological harms caused by stigma, and the negative impact that stigma has on quality of healthcare have been evidenced [51]. Not only is stigma likely to impact an individual’s health and wellbeing, stigma and misinformation about ‘obesity’ also cause barriers to appropriate and timely treatment of many health concerns, not just those that have been linked to weight status [51, 52]. Pont et al. [52] explain that stigma is purported by some as a way to motivate individual weight loss, to tackle the ‘problem’ of obesity; an approach which overlooks the complexity of understanding individual BMI (overstating the control individuals have over it), the contested nature of the links between ‘obesity’ and negative health outcomes, and the negative health outcomes that result from stigma. Interventions which promote stigmatizing messages are likely to have the lowest compliance, whereas interventions which make no reference to obesity at all have been found to be most effective in encouraging health promoting behaviours [53]. By framing stigma as the result of obesity, rather than a problem to challenge, *The Policy* narratively supports individual behaviour change and responsibility, rather than addressing the wider determinants that are necessary to understand the social gradient seen in BMI data and the negative impacts of weight stigma.

Individualising and oversimplifying discourses and evidence around obesity are common within policies and policy networks and perpetuate narratives of individual blame and responsibility for one’s own health status [21, 54]. Stigmatizing policy narratives can detract from structural factors within the SDH which account for many adverse health outcomes and health inequalities that have been linked to obesity [21], which is particularly concerning in the context of policy focussed on children. How obesity is discussed at policy level is critical for public understanding of the topic [53], therefore attention must be paid to the effects of policy narratives and how they can perpetuate stigma.

### The policy and health inequalities

We found several gaps between the proposals in *The Policy* and anticipated outcomes proposed. The fundamental gap identified is that inequality is referred to in the introduction as a crucial element and the conclusion of the policy states that inequality will be reduced as a result of the implementation of the policy and that support is needed for ‘those who need it most’ ([7], p7). However, how this will be achieved in practice is left unclear. Black and minority ethnic families are identified as more likely to be affected by obesity but no explanation for why or how such groups will be affected by the plans is given. Local authorities are encouraged to focus on health inequality, but specific guidance (and support) is unclear. For example, there is recognition of need for greenspace and inequality in access to greenspace, but *The Policy* does not say how it will address this.

Another gap is related to mandatory action or legislation aimed at the early years, a key life stage for understanding the impacts of the SDH and therefore interventions to reduce health inequalities [13, 14]. *The Policy* presents statistics on the prevalence of obesity of children aged 5 years and suggests ‘…*helping to improve the health of our children and give future generations the best possible start in life.*’ ([8], p.4). The reference to early years consists of voluntary food and physical activity guidelines [7] and suggests research is undertaken exploring curriculum development that supports good physical development in the early years, but with no details on the research or proposed timescales [8]. Although there is engagement with early years in the proposed Chapter 3 [9], there is no reference to inequalities.

#### Inequalities and healthy food ‘choices’

*The Policy* has a focus on making healthier food choices without consideration of food insecurity, food bank use and poverty. *The Policy* proposes ideas around ‘choice’ and ‘informed decisions’, for example ‘*I want to see parents empowered to make informed decisions about the food they are buying for their families when eating out*.’ ([8] p.5). However, it lacks consideration of the accessibility of a balanced diet due to: affordability of food, practical considerations on physical cooking equipment and energy costs of preparing and cooking food, skipping meals, needing to use food banks [31, 55] or availability of healthy food options where they live [56].

Food insecurity is associated with poorer diets among children [28], due to limited access to sufficient, varied and healthy foods [57]. Despite this association, there is only one instance where *The Policy* demonstrate an awareness that healthy food is not accessible to all, through a commitment to continue investment in the Healthy Start Scheme. HSS provides pregnant women and families with children under the age of four on low incomes vouchers for milk, fruit, vegetables and vitamins [58]. However, the value of HSS vouchers have remained the same since introduction in 2009 (£3.10 per voucher), despite increasing food prices [59–61]. There is minimal emphasis on the HSS in *The Policy*, evidence of poor implementation of the HSS, and a lack of presence of HSS in the preliminary Chapter 3 of *The Policy* [9]. A 30% decrease in families eligible for the HSS between 2011 and 2018 [30] and recent uptake data demonstrating that less than half of eligible families registered and received HSS vouchers in England [62] bringing the scheme’s effectiveness into question. Reasons for the decline may be due to lack of awareness about the scheme and difficulties with the application process [30, 61].

*The Policy* proposes making healthier choices easier by providing nutritional information through front-of-pack food labelling, implying the ‘problem’ is a lack of information when making food purchasing choices. However, such approaches have the potential to widen health inequalities due to the high level of agency involved [63]. Greater use of UK front-of-pack food labelling by those from more affluent backgrounds, compared with those from disadvantaged backgrounds, is acknowledged [64]. Also, evidence of the effectiveness of front-of-pack labelling is mainly generated using simulated conditions and does not consider financial aspects of purchasing behaviour: a strong driver for those experiencing food and time poverty [65].

### Physical activity and inequalities

In 2019, 24% of children from less affluent backgrounds were classified as physically inactive, in comparison with 12% of children from more affluent backgrounds [66], trends that have been consistently reported since 2015 [67]. The physical activity proposals in *The Policy* centre around advice to schools and funding for cycling and walking initiatives. However, *The Policy* lacks engagement with wider determinants of active travel including environmental constraints, distance from school, and time poverty [68–70] and unmeasured factors found to be associated with cycling including home and social arrangements that facilitate cycling and owning a bike [71]. The proposals do not demonstrate how they are going to target children from less affluent backgrounds to increase physical activity and reduce these inequalities.

In Chapter 2 the money generated by the sugar levy was reported as lower than expected as soft drink manufacturers reformulated products to avoid it. Though a sign of success of the policy, a consequence of this reformulation means less money generated for investment in public health programmes (the PE and sport premium) than was originally estimated, which is not addressed in Chapter 2 or the proposed Chapter 3 [7–9]. The extent to which the premium will support all children, and reduce inequality, through increasing physical activity in school is then brought into question. Questions have been raised about the consistency and accountability of the PE and sport premium in schools, with some aspects of funding lacking clarity about how it will be distributed [72]. As the premium is another initiative that is not targeted based on need, the initiative is unlikely to address inequalities in access to physical activity.

## Conclusions

*The Policy*, described by government as ‘world-leading’ and the first of its kind for children, repeats many of the mistakes of obesity policies that have been shown to be either ineffective or even have adverse effects. The overall problem framing of ‘obesity’ risks reducing the important public health aims to encourage healthy diets and increase opportunities for physical activity (and the physical and mental health benefits of both) for children to weight management, with a focus on particular children, to damaging effect. We have highlighted that individualising of responsibility to respond to systemic factors and structural inequalities may perpetuate damaging narratives and lead to ineffective interventions and ineffective individual treatment. The damaging effects of stigma should not be overlooked, recognising the barriers caused by stigma to opportunities to health promoting behaviours, to positive health outcomes, and to timely and appropriate treatment of health problems. Therefore, careful consideration of the framing of ‘obesity’ is needed from researchers, policy makers (national and local), and public health practitioners as the public health priority of childhood obesity continues to develop and implementation of *The Policy* continues to unfold.

Our approach asked, ‘can the problem be conceptualized differently?’ [33]. From our findings we propose an alternative conceptualisation that obesity rates are illustrative of inequality, as shown by the social gradient, with BMI trends at a population level highlighted in the policy illustrating this. Therefore, rather than ‘obesity’ being the ‘problem’, which we have demonstrated as complex and contested in relation to definition and relationship to health outcomes, we propose that the ‘problem’ to be addressed is inequality. For Chaufan et al. [73], policies that seriously consider the relationship between childhood obesity and socioeconomic inequality, including making poverty and the wider social determinants of health central to their proposals, offer the greatest potential to promote better child health and reduce obesity inequalities. Given the complex and contested relationship between ‘obesity’ and health, it stands that articulating the target policy ‘problem’ as inequality itself (for example, the health gap and access to healthy food and physical activity opportunities) will be more effective in improving health outcomes for all children.

We therefore support a focus on structural inequalities and the social determinants of health (including food security, poverty and environment), rebalancing responsibility away from the individual. The government must work to remove barriers to healthy eating and physical activity particularly those most impacted by health inequalities, regardless of weight, for healthier outcomes for all. Policies must demonstrate how they will tackle inequality and ensure that what is proposed will not widen inequalities through effective engagement with the evidence of the SDH.

## Supplementary Information

**Additional file 1: Table S1.** Key Proposals outlined in *The Policy*, presented independently by chapter.

**Additional file 2: Supplementary Table S2.** Examples of problem representations using Bacchi’s (2009) ‘What’s the problem represented to be?’ approach to analyse *The Policy*

## Data Availability

The qualitative data extracted and analysed during the current study is not publicly available but can be discussed or made available from the corresponding author on reasonable request. All documents analysed are publically available and referenced in this article.

## References

[1] World Health Organization (2017). Report of the commission on ending childhood obesity: implementation plan: executive summary.

[2] Department of Health. The health of the Nation - a strategy for health in England. White Paper. HMSO; 1992.

[3] Jebb SA, Aveyard PN, Hawkes C (2013). The evolution of policy and actions to tackle obesity in England. Obes Rev.

[4] Theis D, White M. Is obesity policy in England fit for purpose? Analysis of government strategies and policies, 1992-2020. Milbank Q. 2013:126–70. 10.1111/1468-0009.12498 PMC7984668 33464689

[5] Croker H, Russell SJ, Gireesh A, Bonham A, Hawkes C, Bedford H, Michie S, Viner RM (2020). Obesity prevention in the early years: a mapping study of national policies in England from a behavioural science perspective. PLoS One.

[6] Davies SC (2019). Time to solve childhood obesity, CMO Special Report.

[7] HM Government. Childhood Obesity: a plan for action. Policy Document. London, 2016. [https://www.gov.uk/government/publications/childhood-obesity-a-plan-for-action] Accessed 10 Dec 2020.

[8] HM Government: Childhood Obesity: a plan for action: Chapter 2. Policy Document. London; 2018. [https://www.gov.uk/government/publications/childhood-obesity-a-plan-for-action-chapter-2] Accessed 10 December 2020.

[9] Department of Health and Social Care: Advancing our health: prevention in the 2020’s. Consultation Document. 2019. [https://www.gov.uk/government/consultations/advancing-our-health-prevention-in-the-2020s/advancing-our-health-prevention-in-the-2020s-consultation-document] Accessed 8 Oct 2020.

[10] Knai C, Petticrew M, Mays N. The childhood obesity strategy. BMJ. 2016:354–i4613. 10.1136/bmj.i4613 27562654

[11] Nobles JD, Summerbell C, Brown T, Jago R, Moore T (2021). A secondary analysis of the childhood obesity prevention cochrane review through a wider determinants of health lens: implications for research funders, researchers, policymakers and practitioners. Int J Behav Nutr Phys Act.

[12] Black D, Morris JN, Smith C, Townsend P, Davidson N, Whitehead M (1980). The black report: inequalities in health.

[13] Marmot Review (2010). Fair society, healthy lives: strategic review of health inequalities in England post-2010.

[14] Marmot M, Allen J, Boyce T, Goldblatt P, Morrison J (2020). Health equity in England: The Marmot Review 10 years on.

[15] MacKenzie M, Collins C, Connolly J, Doyle M, McCartney G (2017). Working-class discourses of politics, policy and health: ‘I don’t smoke; don’t drink. The only thing wrong with me is my health’. Policy Polit.

[16] Wistow J, Blackman T, Byrne D, Wistow G (2015). Studying health inequalities: an applied approach.

[17] Chung A, Backholer K, Wong E, Palermo C, Keating C, Peeters A (2016). Trends in child and adolescent obesity prevalence in economically advanced countries according to socioeconomic position: a systematic review. Obes Rev.

[18] El-Sayed AM, Scarborough P, Galea S (2012). Unevenly distributed: a systematic review of the health literature about socioeconomic inequalities in adult obesity in the United Kingdom. BMC Public Health.

[19] NHS Digital. National Child Measurement Programme, England 2019/2021 School Year, Official National Statistics. 2020. [https://digital.nhs.uk/data-and-information/publications/statistical/national-child-measurement-programme/2019-20-school-year] Accessed 5 Jan 2021.

[20] Kriznik NM, Kinmonth AL, Ling T, Kelly MP (2018). Moving beyond individual choice in policies to reduce health inequalities: the integration of dynamic with individual explanations. J Public Health.

[21] Medvedyuk S, Ali A, Raphael D (2018). Ideology, obesity and the social determinants of health: a critical analysis of the obesity and health relationship. Crit Public Health.

[22] Nobles J, Christensen A, Butler M, Radley D, Pickering K, Saunders J, Weir C, Sahota P, Gately P (2019). Understanding how local authorities in England address obesity: A wider determinants of health perspective. Health Policy.

[23] Brown T, Moore T, Hooper L, Gao Y, Zayegh A, Ijaz S, Elwenspoek M, Foxen SC, Magee L, O'Malley C, Waters E, Summerbell CD (2019). Interventions for preventing obesity in children. Cochrane Database Syst Rev.

[24] Hillier-Brown FC, Bambra CL, Cairns-Nagi JM, Kasim A, Moore HJ, Summerbell CD (2014). A systematic review of the effectiveness of individual, community and societal level interventions at reducing socioeconomic inequalities in obesity amongst children. BMC Public Health.

[25] Food Foundation. New evidence of child food insecurity in the UK. 2017. [https://foodfoundation.org.uk/new-evidence-of-child-food-insecurity-in-the-uk/] Accessed 6 Oct 2020.

[26] Holding E, Fairbrother H, Griffin N, Wistow J, Powell K, Summerbell C (2021). Exploring the local policy context for reducing health inequalities in children and young people: an in depth qualitative case study of one local authority in the North of England, UK. BMC Public Health.

[27] Bambra C, Riordan R, Ford J, Matthews F (2020). The COVID-19 pandemic and health inequalities. J Epidemiol Community Health.

[28] Power M, Pybus KJ, Pickett KE, Doherty B (2021). “The reality is that on Universal Credit I cannot provide the recommended amount of fresh fruit and vegetables per day for my children”: Moving from a behavioural to a systemic understanding of food practices. Emerald Open Res.

[29] The Trussell Trust (2020). End of year stats.

[30] Crawley H, Dodds R: The UK healthy start scheme. What happened? What next?. First Steps Nutrition Trust; 2018 [https://static1.squarespace.com/static/59f75004f09ca48694070f3b/t/5b8e2d0e575d1f6f1e5d2dcd/1536044307456/Healthy_Start_Report_for_web.pdf] Accessed 5 Oct 2020.

[31] Select Committee on Food, Poverty, Health and the Environment. Hungry for change: fixing the failures in food. 2020. [https://committees.parliament.uk/publications/1762/documents/17092/default/] Accessed 8 Oct 2020.

[32] Pawson R, Tilley N (2004). Realist evaluation.

[33] Bacchi C (2009). Analysing policy: What’s the problem represented to be?.

[34] Bacchi C (2016). Problematizations in health policy: questioning how “problems” are constituted in policies. SAGE Open.

[35] Smith B, McGannon KR (2018). Developing rigor in qualitative research: problems and opportunities within sport and exercise psychology. Int Rev Sport Exerc Psychol.

[36] Chapman P, Lindsey I, Dodd-Reynolds C, Oliver E, Summerbell C (2020). Targeting childhood obesity through primary schools: reviewing alignment amongst English policies for physical activity and healthy eating. Child Adolesc Obes.

[37] Lakerveld J, Mackenbach J (2017). The upstream determinants of adult obesity. Obes Facts.

[38] L’Hôte E, Fond M, Volmert A (2018). Communicating about obesity: a frameworks strategic report.

[39] Baum F, Fisher M (2014). Why behavioural health promotion endures despite its failure to reduce health inequities. Sociol Health Illn.

[40] Correia JC, Somers F, Golay A, Pataky Z (2020). Obesity : eat less and move more ? Not so easy. Rev Med Suisse.

[41] Gillborn S, Rickett B, Muskett T, Woolhouse M (2020). Apocalyptic public health: exploring discourses of fatness in childhood ‘obesity’policy. J Educ Policy.

[42] Evans B, Colls R (2009). Measuring fatness, governing bodies: the spatialities of the body mass index (BMI) in anti-obesity politics. Antipode.

[43] Evans B, Colls R, Hoerschelmann K (2011). ‘Change4Life for your kids’: embodied collectives and public health pedagogy. Sport Educ Soc.

[44] Salas XR, Forhan M, Caulfield T, Sharma AM, Raine K (2017). A critical analysis of obesity prevention policies and strategies. Can J Public Health.

[45] Chiappetta S, Sharma AM, Bottino V, Stier C (2020). COVID-19 and the role of chronic inflammation in patients with obesity. Int J Obes.

[46] Simmonds M, Llewellyn A, Owen CG, Woolacott N (2016). Predicting adult obesity from childhood obesity: a systematic review and meta-analysis. Obes Rev.

[47] Umer A, Kelley GA, Cottrell LE, Giacobbi P, Innes KE, Lilly CL (2017). Childhood obesity and adult cardiovascular disease risk factors: a systematic review with meta-analysis. BMC Public Health.

[48] Lloyd LJ, Langley-Evans SC, McMullen S (2012). Childhood obesity and risk of the adult metabolic syndrome: a systematic review. Int J Obes.

[49] Richardson AK (2012). Investing in public health: barriers and possible solutions. J Public Health.

[50] Salas XR (2015). The ineffectiveness and unintended consequences of the public health war on obesity. Can J Public Health.

[51] Rubino F, Puhl RM, Cummings DE, Eckel RH, Ryan DH, Mechanick JI, Nadglowski J, Salas XR, Schauer PR, Twenefour D, Apovian CM (2020). Joint international consensus statement for ending stigma of obesity. Nat Med.

[52] Pont SJ, Puhl R, Cook SR, Slusser W (2017). Stigma experienced by children and adolescents with obesity. Pediatrics.

[53] Puhl R, Peterson JL, Luedicke J (2013). Fighting obesity or obese persons? Public perceptions of obesity-related health messages. Int J Obes.

[54] Thibodeau PH, Perko VL, Flusberg SJ (2015). The relationship between narrative classification of obesity and support for public policy interventions. Soc Sci Med.

[55] Harvey K (2016). “When I go to bed hungry and sleep, I’m not hungry”: Children and parents' experiences of food insecurity. Appetite.

[56] Colls R, Evans B (2014). Making space for fat bodies?: A critical account of ‘the obesogenic environment’. Prog Hum Geogr.

[57] Skafida V, Treanor MC (2014). Do changes in objective and subjective family income predict change in children’s diets over time? Unique insights using a longitudinal cohort study and fixed effects analysis. J Epidemiol Community Health.

[58] Healthy Start [https://www.healthystart.nhs.uk/] Accessed 8 Oct 2020.

[59] Department for Environment Food and Rural Affairs. Food statistics in your pocket: prices and expenditure. [https://www.gov.uk/government/publications/food-statistics-pocketbook/food-statistics-in-your-pocket-prices-and-expenditure#uk-retail-price-changes-by-food-group-2009-to-2019] Accessed 6 Oct 2020.

[60] Lucas PJ, Jessiman T, Cameron A (2015). Healthy start: the use of welfare food vouchers by low-income parents in England. Soc Policy Soc.

[61] McFadden A, Green JM, Williams V, McLeish J, McCormick F, Fox-Rushby J, Renfrew MJ (2014). Can food vouchers improve nutrition and reduce health inequalities in low-income mothers and young children: a multi-method evaluation of the experiences of beneficiaries and practitioners of the Healthy Start programme in England. BMC Public Health.

[62] Healthy Start Uptake Data [https://www.healthystart.nhs.uk/healthy-start-uptake-data/] Accessed 5 Oct 2020.

[63] Adams J, Mytton O, White M, Monsivais P (2016). Why are some population interventions for diet and obesity more equitable and effective than others? The role of individual agency. PLoS Med.

[64] Department of Health and Social Care (2020). Tackling obesity: empowering adults and children to live healthier lives.

[65] Puddephatt JA, Keenan GS, Fielden A, Reaves DL, Halford JC, Hardman CA (2020). ‘Eating to survive’: A qualitative analysis of factors influencing food choice and eating behaviour in a food-insecure population. Appetite.

[66] Sport England (2020). Active lives data tables.

[67] Fuller E, Mindell J, Prior G. Health survey for England 2015. NHS Digital; 2016.

[68] Ahern SM, Arnott B, Chatterton T, de Nazelle A, Kellar I, McEachan RR (2017). Understanding parents’ school travel choices: a qualitative study using the theoretical domains framework. J Transp Health.

[69] Kalenkoski CM, Hamrick KS (2013). How does time poverty affect behavior? A look at eating and physical activity. Appl Econ Perspect Policy.

[70] Stewart O, Moudon AV, Claybrooke C (2012). Common ground: eight factors that influence walking and biking to school. Transp Policy.

[71] McKay A, Goodman A, Van Sluijs E, Millett C, Laverty AA (2020). Cycle training and factors associated with cycling among adolescents in England. J Transp Health.

[72] Lindsey I (2020). Analysing policy change and continuity: physical education and school sport policy in England since 2010. Sport Educ Soc.

[73] Chaufan C, Yeh J, Ross L, Fox P (2015). You can’t walk or bike yourself out of the health effects of poverty: active school transport, child obesity, and blind spots in the public health literature. Crit Public Health.

