# Supplementary material for: A critique of the English national policy from a social determinants of health perspective using a realist and problem representation approach: the ‘Childhood Obesity: a plan for action’ (2016, 2018, 2019)
Source: BMC Public Health. 2021 Dec 14;21:2284. doi: 10.1186/s12889-021-12364-6 (PMC8669235; doi:10.1186/s12889-021-12364-6)
Supplement: Supplementary file 1 — Additional file 1: Table S1. Key Proposals outlined in The Policy, presented independently by chapter. [file 12889_2021_12364_MOESM1_ESM.docx]

Supplementary Information

Table S1: Key Proposals outlined in *The Policy*, presented independently by chapter.

| Chapter 1 | Chapter 2 | Chapter 3 (**consultation document**) |
| --- | --- | --- |
| **Overarching proposed aims** | |  |
| - Reduce number of overweight and obese children within 10 years - Reducing obesity will save lives and reduce inequalities | - Halve childhood obesity rates by 2030 - Reduce gap in obesity between children from most and least deprived areas by 2030 |  |
| **Sugar reduction** | |  |
| - Soft drinks industry levy (SDIL) (technical details in 2016, legislate Finance bill in 2017) - Reduce 20% sugar in selected products by 2020, 5% reduction in first year | - Consider introduction of sugary milk drinks in SDIL (2020 review) - Consult on introducing legislation to ban sale of energy drinks to children (2018) - Further use of tax system to promote healthy food | - End sale of energy drinks to children under age of 16 years. - May extend the SDIL to sugary milk drinks |
| **Calorie reduction** | |  |
| - Calorie reduction programme (2017) - Update nutrient profile model to reflect government dietary guidelines - Review opportunities for clearer food labelling - Encourage business innovation to create healthier products - Technology to create apps to inform eating decisions and develop weight management support for children and families | - Consult and introduce calorie labelling legislation on out of home sector (2018) - Explore clearer food labelling | - Consult of front-of-package nutritional labelling |
| **Salt reduction** | | |
|  |  | - Publish revised salt reduction targets |
| **Healthy Start Scheme (HSS)** | |  |
| - Re-commit to HSS | - Consult on plans to use HSS vouchers (2018) |  |
| **Schools** | |  |
| **Physical activity:**   - Primary PE/Sport premium (from the revenue of SDIL) - Advise schools with spending of the premium (2017/18) - Online tool to help schools plan at least 30 minutes of PA per day - National governing bodies to offer high quality sport programmes to every primary school (2017) - £300m to increase children walking to school and Bikeability training.     **Healthy eating:**   - Creating healthy rating scheme for primary schools- Ofsted inspected, involve parents in rating process, run annual competition to recognise innovative schools (2017) - Campaign to encourage all schools to commit to School Food Standards - Invest in healthy breakfast clubs (from the revenue of SDIL) - Ofsted to conduct thematic review on obesity in schools (2017) | **Physical activity:**   - Review how the least active children are being engaged in physical activity in and around the school day. - Every primary school to adopt an active mile initiative (e.g Daily Mile). - £1.6million to support cycling and walking to school (2018/19)     **Healthy eating:**   - Update school food guidance - Consult on strengthening the nutrition standards in the Government Buying Standards for Food and Catering Services (GBSF), to bring them into line with the latest scientific dietary advice (2018)      - Ofsted inspection framework - to include healthy eating and PA across curriculum (2019) | **Physical activity:**   - New PA guidelines to be published |
| **Early years** | | |
| - Revised menus for early years settings to meet government guidelines (2016) - Awareness campaign for PA guidelines (2017) - Update Early Years Foundation Stage framework to reference to the PA guidelines. | - Ofsted to research what a curriculum that supports good physical development in the early years looks like - Review scope for reformulation of product ranges aimed exclusively at babies and young children (2019) | - Commission an infant feeding survey- information on breastfeeding and use of food/drinks other than breastmilk - Public health England to publish guidelines for industry on nutritional content of baby food and drinks - Improve marketing and labelling of infant food |
| **Advertising and Promotions** | |  |
|  | - Watershed on TV advertising of HFSS products and similar protection for children viewing adverts online (2018) - Consider whether legislation is necessary for online advertising rules. - Ban price promotions on unhealthy food and drink in retail and out of home sector (consult in 2018) - Ban promotion of unhealthy food and drink by location (e.g. checkouts in supermarkets) (consult in 2018) |  |
| **Local areas** | |  |
| - Encourage local authorities and local government to adopt Government Buying Standards for Food and Catering Services (GBSF) - Training/resources to health care professionals to discuss and provide support around weight related issues | - Trailblazer programme with LA partners - Resources (guidance and training) to support LA in creating ‘healthy food environments’ - Define standards to demonstrate “good” green infrastructure  (2019) - Provide health care professionals with training/tools to support families - Consult on strengthening the nutrition standards in the GBSF (2018) | - Develop approaches to improve quality of brief advice given on health issues in general practice - Review current digital weight management products/services, continue development of ‘Our Family Health’ - NCMP- explore how it can be embedded in digital approaches and how data can be used to enable families access to support - Working across government to improve PA opportunities (e.g. LA planning to promote active lifestyles). |
