# Supplementary material for: A critique of the English national policy from a social determinants of health perspective using a realist and problem representation approach: the ‘Childhood Obesity: a plan for action’ (2016, 2018, 2019)
Source: BMC Public Health. 2021 Dec 14;21:2284. doi: 10.1186/s12889-021-12364-6 (PMC8669235; doi:10.1186/s12889-021-12364-6)
Supplement: Supplementary file 2 — Additional file 2: Supplementary Table S2. Examples of problem representations using Bacchi’s (2009) ‘What’s the problem represented to be?’ approach to analyse The Policy [file 12889_2021_12364_MOESM2_ESM.docx]

| What’s the ‘problem’ represented to be? | How has this representation of the ‘problem’ come about? | What assumptions underpin the representation of ‘the problem’? | What effects are produced by this representation of the ‘problem’? | What is left unproblematic? | How might the policy response differ? Can the ‘problem’ be conceptualised differently? |
| --- | --- | --- | --- | --- | --- |
| - Prevalence of obesity in children growing - Children from low income families more likely to be overweight or obese - Obesity as a health problem - Food and drink choices - High levels of sugar in food/drinks - Lack of parental knowledge and understanding of nutrition - Children not sufficiently active during school | - Cost to NHS of obesity linked to co-morbidities in adults (preventative measures) - Obesity rates higher in children from low-income households and areas of higher socio-economic deprivation - Wider changes in public health policy towards behaviour change and individual focus | - That universal proposals to reduce obesity will narrow the health inequalities gap - NCMP (based on BMI) as an accurate measure of child health - Lack of understanding of nutrition within families - Accessibility of the proposed initiatives (e.g. that families will have resources to enact proposals) | - Focus on ‘problem’ individuals rather than targeting structural inequalities - Contribution to stigma and oversimplifying of links between health and body size (linking to negative effects of stigma) | - Stigma as a reason to target obesity, rather than targeting stigma - Poverty as reason to tackle obesity, rather than needing to tackle poverty - Absence of the impact of austerity on health budgets (putting the focus on individual responsibility rather than s service provision issue) | - Improving health (through healthy eating and physical activity) rather than focus on weight / reducing obesity - Addressing barriers to healthy eating and physical activity for all children, regardless of weight - Focus on structural inequalities and the social determinants of health, including poverty, food security and environmental factors. |

Supplementary Table 2 (cited as Table S2 in the text)

Examples of problem representations using Bacchi’s (2009) ‘What’s the problem represented to be?’ approach to analyse *The Policy*
